# Food Insecurity, Healthcare Utilization, and Healthcare Expenditures: A Longitudinal Cohort Study

**DOI:** 10.3389/ijph.2023.1605360

**Published:** 2023-07-26

**Authors:** Hwi Choe, Tae-Young Pak

**Affiliations:** Department of Consumer Science and Convergence Program for Social Innovation, Sungkyunkwan University, Seoul, Republic of Korea

**Keywords:** food insecurity, poverty, hunger, healthcare utilization, healthcare expenditure

## Abstract

**Objective:** This study examines the longitudinal association between household food insecurity and healthcare utilization and expenditure.

**Methods:** A multi-wave longitudinal cohort study was conducted using the 2008–2019 and 2021 waves of the Korean Welfare Panel Study. The baseline data included participants aged ≥19 years with valid responses to the food insecurity and healthcare questionnaires in the 2008 wave (*n* = 12,166). Healthcare outcomes encompassed outpatient visits, inpatient admissions, days hospitalized, and personal healthcare expenditure. Random effects Poisson and linear regressions were estimated.

**Results:** Severe food insecurity was associated with a higher incidence rate of outpatient visits (IRR, 1.14; 95% CI, 1.12–1.17), days of hospitalization (IRR, 1.18; 95% CI, 1.13–1.22), and inpatient admissions (IRR, 1.40; 95% CI, 1.18–1.65). Moderate food insecurity was associated with 10.4% ( β = −0.11; 95% CI, −0.14 to −0.07) or 238,276 KRW reductions in personal healthcare expenditures in the subsequent year.

**Conclusion:** Household food insecurity was linked to increased healthcare utilization and reduced personal healthcare expenditure among Korean adults. Our findings present opportunities to identify target populations for healthcare policies and interventions.

## Introduction

Food insecurity occurs when households are unable to acquire nutritionally adequate and safe food due to insufficient financial means and resources. Between 2019 and 2021, more than 2.7 million or 5.3% of the Korean population reported experiencing food insecurity in the preceding 12 months [1]. Approximately 0.7% experienced 24-h periods of caloric deprivation, a condition referred to as “severe food insecurity” [1]. Food insecurity constitutes a complex social and economic problem with significant health and wellbeing implications [2]. Individuals in food insecure households face tradeoffs between food and other necessities, including medication, housing, and utilities [3, 4].

The growing evidence points to food insecurity as a strong predictor of preventable chronic conditions, as well as poor physical and mental health [5–11]. Food insecurity can lead to adverse health outcomes through several mechanisms, including nutritionally inadequate diets, stress and anxiety, challenges in managing chronic conditions, and medication non-adherence [5, 11–13]. These factors jointly contribute to higher mortality rates and premature deaths among food insecure populations in the United States [14] and Canada [15].

Prior research has documented the challenges that food insecure households face when obtaining medical services; they are more susceptible to illnesses and have greater medical needs, yet they often delay care when it is needed [16–18]. Some studies have shown that food insecurity leads to increased medical expenditure and greater use of acute care services among children [19, 20], older adults [17, 21], and individuals with chronic conditions [22]. On the other hand, several studies have reported reduced outpatient visits and lower access to modern healthcare in food insecure populations [20, 23, 24]. In South Korea (Korea, hereafter), where healthcare provision is universal, food insecure individuals can access primary and routine care at an affordable rate, but they may encounter financial barriers to advanced medical services that require significant co-payments or out-of-pocket expenses [25, 26]. Consequently, they may opt for cheaper treatment alternatives instead of the suggested treatment plan, and be unable to receive timely care for their medical conditions. The co-payment burden can result in delayed or insufficient healthcare utilization, which can further exacerbate health problems [27, 28].

Conceptual models suggest that food insecurity can have both positive and negative effects on medical service use. The neoclassical model of household production [29] predicts that household consumption, including medical care, is restricted by resource constraints. Healthier diets generally cost more, and food insecure households may sacrifice basic necessities to secure food-related budgets [3]. This sacrifice may include limiting budgets for healthcare, leading food insecure individuals to forgo necessary healthcare and medications. An alternative framework by Andersen and Newman proposes that healthcare utilization is determined by three factors: a) predisposing component (one’s predisposition to healthcare use); b) enabling component (one’s ability to access healthcare); and c) illness level (one’s need for healthcare services) [30]. Food insecurity is considered a predisposing factor for healthcare use, as increased vulnerability among food-insecure individuals may generate demand for medical services [23]. The comorbidities associated with food insecurity may induce the need for healthcare, resulting in a positive association between food insecurity and healthcare utilization [19–22, 31–37].

Currently, there exists limited research on how food insecure Korean adults address the dual challenges of healthcare access and food insecurity. In this study, we utilized data from the Korean Welfare Panel Study to examine the longitudinal associations between food insecurity and subsequent healthcare utilization and expenditure in a nationally representative cohort of Korean adults aged ≥19 years. This study improves upon prior research by a) employing multi-wave longitudinal data with 12 years of follow-up, b) controlling for employment, income, and consumption variables that may confound the effect of food insecurity, and c) examining a healthcare system characterized by high co-payments and out-of-pocket expenses for medical services [25]. We hypothesize that food insecurity is associated with greater healthcare use but lower out-of-pocket medical spending among Korean adults.

### Healthcare Delivery in South Korea

The National Health Insurance (NHI) in Korea is a critical component of the nation’s healthcare delivery, providing access to medical services for the general population [38, 39]. The NHI was established in 1977, initially mandating health insurance coverage for businesses with more than 500 employees. By 1989, it expanded to include all citizens, becoming a universal healthcare program. In 2000, the Korean government consolidated 370 insurance funds and unions into a single insurer, the National Health Insurance Corporation, to manage mandatory participation and operation of the NHI [40]. Korean citizens can enroll in the NHI through two channels: the employer-provided program and the locally provided program [39]. The employer-provided program serves employees whose insurance premiums are partially paid by their employers. The locally provided program caters to those who are neither employed by others nor family members of policy-holding employees.

The NHI is comprehensive in its coverage, yet it falls short in certain areas. While the benefits package includes most high-probability inpatient and outpatient services, it excludes low-probability, high-cost medical services used to treat severe health conditions [41]. High out-of-pocket cost burdens are also imposed on covered services. On average, a Korean citizen pays 1,065 USD or 30.3% of their annual healthcare expenses out-of-pocket [42]. The co-payment rates vary depending on the types of care and provider characteristics, ranging from 30% for outpatient services at local clinics to 50%–60% for outpatient services and doctors’ fees at tertiary care hospitals [26]. The NHI has gradually expanded to alleviate co-payment burden for severe conditions [43]. Despite the program’s continued expansion, medical examinations and services essential for survival remain not fully covered by the NHI, thereby creating a financial barrier to healthcare utilization [44].

As low-income individuals carry greater health risks, they tend to spend a disproportionately large portion of their financial resources on outpatient care and medications [45]. Studies have demonstrated that catastrophic health expenditure is a significant predictor of poverty among marginalized households in Korea [45–47]. Furthermore, researchers found that high out-of-pocket medical expenses deter these households from seeking necessary medical care, which can exacerbate their health conditions and widen the health gap between wealthier and poorer households [48]. The co-payment structure of the NHI poses a greater financial burden on low-income families with severely ill members [46], and these costs can rapidly accumulate over the course of treatments. In the United States, such households often cut back on other necessities such as food and housing [49], exhaust savings [50], or even fall into a medical debt trap [51], although evidence is not concrete in the Korean context.

## Methods

### Data Source and Study Sample

This study utilized multi-wave data from the Korean Welfare Panel Study (KoWePS). The KoWePS is a longitudinal and nationally representative survey of community-dwelling Korean adults, administered by the Korean Institute of Social and Health Affairs and Seoul National University. The initial sample was drawn from the Census using a two-stage stratified cluster sampling design and has been tracked every year since 2006. The KoWePS includes data on demographic background, household assets, welfare use, health status, subjective wellbeing, and healthcare utilization. Food insecurity was first assessed in the 2008 survey and has been recorded annually since then. The study sample includes KoWePS participants aged ≥19 years who provided valid responses to the food insecurity and healthcare questionnaires in 2008 (*N* = 12,166), and their follow-up measurements in the 2009–2019 and 2021 surveys. The participants in the baseline sample were tracked for an average of 10 years, allowing us 122,241 observations (Supplementary Table S1). The Institutional Review Board at Sungkyunkwan University exempted this study from human subject review as it used de-identified data.

### Food Insecurity

Food insecurity was assessed using the Korean Household Food Security Survey (KHFSS), which inquires household food access over the last 12 months. The measurement items consist of (a) “In the preceding year, because of economic hardship, I did not have enough money to buy food even when I was out of food”; (b) “In the preceding year, because of economic hardship, I did not have enough money to have balanced meals (in sufficient amounts of various diets)”; (c) “In the preceding year, have any adults in your household reduced the amount of meals or skipped meals because there was not enough money to buy food?” (d) (If answered yes) “How often did this happen?”; (e) “In the preceding year, have you eaten less than you felt you should because there was not enough money to buy food?”; (f) “In the preceding year, have you been unable to eat even when you were hungry because there was not enough money to buy food?”. For items (a) and (b), the responses “often” and “sometimes” were coded as 1, and “never” was coded as 0. For items (c), (e), and (f), “yes” was scored as 1 and “no” as 0. For item (d), the responses “almost every month” or “some months but not every month” were scored as 1 and “1 or 2 months” was scored as 0. The response “don’t know” and refusal to participate were considered missing and excluded from the sample. The summed responses range from 0 to 6, indicating the degree of food insecurity. Indicators of food security (total score ≤1), moderate food insecurity (total score 2–4), and severe food insecurity (total score ≥5) were defined according to thresholds validated in previous literature (Table 1). The food security category includes households with no limitations in fulfilling their food preferences or dietary needs. Moderate food insecurity pertains to households that report anxiety about food access or experience reduced quality of diets. Finally, severe food insecurity indicates households that undergo disrupted eating patterns and reduced food intake due to financial hardship.

**TABLE 1:** Response to the Korean household food security survey (Korean Welfare Panel Study, South Korea, 2008).

Affirmative responses	% of 2008 wave
0 (food security)	84.8
1 (food security)	9.2
2 (moderate food insecurity)	3.7
3 (moderate food insecurity)	0.8
4 (moderate food insecurity)	0.6
5 (severe food insecurity)	0.6
6 (severe food insecurity)	0.5

### Healthcare Utilization and Expenditures

Measures of healthcare utilization include the number of outpatient hospital visits, inpatient hospital admissions, and days spent as a hospital patient between 1 January and 31 December in the preceding year. Participants were instructed to report their access to care and services provided by legally accredited medical institutions and not to include preventive care. They were further instructed that visits to multiple departments within a hospital should be counted as one. The responses were reported as counts (outpatient visits, inpatient admissions) and days (inpatient days). Those with no healthcare utilization were instructed to fill in “00” as their response.

Personal healthcare expenditure is defined as the mean monthly spending on healthcare services in the preceding year. This includes out-of-pocket expenditures for inpatient and outpatient services, dental care, surgeries (including dental and plastic surgery), medications, nursing care, preventive care, dietary supplements, and miscellaneous healthcare products (e.g., corrective lenses and knee guards). The response was reported in 10,000 KRW (equivalent to approximately 8 USD) and converted to 2020 KRW using the Korean Consumer Price Index. This variable was transformed using $\ln\left(x+1\right)$ to address the skewness of the distribution.

### Covariates

Covariates were selected based on the literature and include demographic and socioeconomic variables that might be correlated with food insecurity or healthcare utilization. These include indicators of age (in years), sex (female or male), educational background (middle school or less; high school graduate; college graduate), marital status (not in a marital relationship; currently married), number of household members, region of residence (rural area, urban area, metropolitan city), chronic conditions (high blood pressure, diabetes, cancer, heart disease, stroke, arthritis), self-rated health (very poor, poor, fair, good, very good), disability, employment status (employed, self-employed, retired), yearly household income (in quintiles), monthly household consumption (in quintiles), and year of survey. The category “not in a marital relationship” includes participants who were separated from their partners and those who were divorced, widowed, or never married. The “retired” category of employment status includes individuals who were retired, unemployed, and not in the labor market. Disability is determined by whether a participant is registered as disabled for welfare claiming purposes. Household income and consumption are re-scaled into 2020 KRW and collapsed into quintiles.

### Statistical Analysis

Our main empirical specification comprises individual random effects Poisson regression (outpatient visits, inpatient admissions, days hospitalized) and individual random effects linear regression (log of healthcare expenditures). We first estimated unadjusted regression models, including only the food insecurity indicator and year of survey dummies. The association between food insecurity and healthcare outcomes was then adjusted for demographic factors, health status, and socioeconomic characteristics in separate analyses. Our preferred specification is a fully adjusted model that controls for all the covariates. For the Poisson regression results, we obtained incidence rate ratios (IRR) by exponentiating the coefficient estimates. Across all regressions, the adjusted IRR and adjusted beta coefficients were reported, along with their 95% confidence intervals (CI). Coefficients with p <0.05 for a two-sided test were considered statistically significant. All analyses were performed using Stata/SE 16.1. The estimated results were weighted using the sampling weights provided by the KoWePS. Data cleaning and analyses were performed from 1 February to 27 March 2022.

## Results

The baseline sample includes 12,166 participants aged ≥19 years in 2008 (Table 2). The sample consists of 6,631 women (54.5%), 5,535 (45.5%) men, 3,276 (26.9%) college graduates, 8,007 (65.8%) married individuals, and 5,311 (43.7%) metropolitan city residents. A total of 5,150 participants (42.3%) rated their overall health as good, and 1,511 (12.4%) rated their health as great. Overall, 739 participants (6.0%) lived in food insecure households; of these, 609 (5.0%) households were moderately and 130 (1.1%) were severely food insecure. The mean number of outpatient visits was 15.4 (27.1%), and the mean numbers of days spent hospitalized and inpatient admissions were 2.85 (16.5%) and 0.17 (0.64%), respectively. Comparing healthcare variables by food insecurity shows that households with food insecurity reported more outpatient visits, days spent hospitalized, and inpatient admissions but were less inclined to spend on healthcare.

**TABLE 2:** Baseline sample characteristics by food insecurity (Korean Welfare Panel Study, South Korea, 2008).

	No. of respondents (%) or mean (SD)			
Variables	Food secure (*n* = 11,427)	Moderately food insecure (*n* = 609)	Severely food insecure (*n* = 130)	All (*n* = 12,166)
Outpatient visits	15.0 (26.7)	21.3 (31.1)	21.9 (31.0)	15.4 (27.1)
Inpatient days	2.79 (16.5)	3.75 (16.1)	4.23 (13.1)	2.85 (16.5)
Inpatient admissions	0.16 (0.63)	0.24 (0.75)	0.22 (0.57)	0.17 (0.64)
Healthcare expenditures (monthly)^a^	16.5 (27.0)	11.1 (23.8)	6.8 (9.8)	16.1 (26.8)
Age (yrs)	50.4 (17.7)	54.7 (17.7)	57.7 (17.0)	50.7 (17.7)
Sex				
Female	6,207 (54.3)	349 (57.1)	75 (58.4)	6,631 (54.5)
Male	5,220 (45.7)	260 (42.9)	55 (41.6)	5,535 (45.5)
Educational level				
Middle school or less	4,902 (42.9)	368 (60.7)	88 (65.8)	5,358 (44.0)
High school graduate	3,334 (29.2)	166 (26.8)	32 (26.8)	3,532 (29.0)
College graduate	3,191 (27.9)	75 (12.5)	10 (7.4)	3,276 (26.9)
Marital status				
Not in marital relationship^b^	3,758 (32.9)	321 (52.4)	80 (61.7)	4,159 (34.2)
Currently married	7,669 (67.1)	288 (47.6)	50 (38.3)	8,007 (65.8)
Number of household members	3.05 (1.28)	2.63 (1.38)	2.15 (1.34)	3.02 (1.29)
Region of residence				
Rural area	2,844 (24.9)	115 (18.9)	26 (20.0)	2,985 (24.5)
Urban area	3,669 (32.1)	159 (26.1)	42 (32.3)	3,870 (31.8)
Metropolitan city	4,914 (43.0)	335 (55.0)	62 (47.7)	5,311 (43.7)
Chronic conditions				
High blood pressure	1,398 (12.2)	81 (13.3)	27 (20.8)	1,506 (12.4)
Diabetes	523 (4.6)	46 (7.6)	10 (7.7)	579 (4.8)
Cancer	134 (1.2)	12 (2.0)	0 (0)	146 (1.2)
Heart disease	218 (1.9)	13 (2.1)	3 (2.3)	234 (1.9)
Stroke	169 (1.5)	17 (2.8)	5 (3.8)	191 (1.6)
Arthritis	1,253 (11.0)	95 (15.6)	21 (16.2)	1,369 (11.3)
Self-rated health				
Very poor	352 (3.1)	65 (10.7)	27 (20.8)	444 (3.6)
Poor	2,293 (20.1)	212 (34.8)	52 (40.0)	2,557 (21.0)
Fair	2,348 (20.5)	130 (21.3)	26 (20.0)	2,504 (20.6)
Good	4,970 (43.5)	161 (26.4)	19 (14.6)	5,150 (42.3)
Very good	1,464 (12.8)	41 (6.7)	6 (4.6)	1,511 (12.4)
Disability	1,094 (9.6)	114 (18.7)	31 (23.8)	1,239 (10.2)
Employment status				
Employed	4,208 (36.8)	178 (29.2)	28 (21.5)	4,414 (36.3)
Self employed	1,812 (15.9)	59 (9.7)	12 (9.2)	1,883 (15.5)
Retired	5,407 (47.3)	372 (61.1)	90 (69.2)	5,869 (48.2)
Household income (yearly)	4,445 (4,317)	1,910 (1,472)	1,101 (1,131)	4,282 (4,247)
Household consumption (monthly)	329 (267)	171 (123)	123 (78)	319 (263)

Abbreviations: SD, standard deviation; n, number of observations.

^a^ Monetary values expressed in 10k KRW.

^b^ Includes separated, divorced, widowed, and never married.

Table 3 presents the results of random effects Poisson and linear regression analyses for healthcare utilization and expenditure (full results in Supplementary Tables S2–S5). The baseline models indicate that severe food insecurity was associated with increased incidence rates of outpatient visits (IRR, 1.16; 95% CI, 1.14–1.18), days of hospitalization (IRR, 1.16; 95% CI, 1.11–1.20), inpatient admissions (IRR, 1.52; 95% CI, 1.28–1.80), and lower healthcare expenditure (*β* = −0.09; 95% CI, −0.18–0.00). Moderate food insecurity was associated with higher inpatient admission incidence rates (IRR, 1.12; 95% CI, 1.03–1.21) and lower healthcare expenditure (*β* = −0.15; 95% CI, −0.19 to −0.11). Upon controlling for demographic factors, severe food insecurity exhibited positive associations with increased outpatient visits (IRR, 1.15; 95% CI, 1.13–1.17), inpatient visits (IRR, 1.12; 95% CI, 1.08–1.17), and inpatient admissions (IRR, 1.41; 95% CI, 1.19–1.67). The health status-adjusted models show estimated IRRs of 1.15 (95% CI, 1.13–1.17), 1.12 (95% CI, 1.08–1.16), and 1.30 (95% CI, 1.10–1.54) for the associations between severe food insecurity and healthcare utilization variables.

**TABLE 3:** Association of food insecurity at *t*–1 with healthcare utilization and expenditures at *t*, random effects Poisson and linear regression results (Korean Welfare Panel Study, South Korea, 2008–2019, 2021).

	Outpatient visits		Inpatient days		Inpatient admissions		Healthcare expenditures	
	IRR (95% CI)^a^	*P* val.	IRR (95% CI)^a^	*P* val.	IRR (95% CI)^a^	*P* val.	Adjusted β (95% CI)^b^	*P* val.
Baseline model^c^								
Severe food insecurity (*t*–1)	1.16 (1.14, 1.18)	<0.001	1.16 (1.11, 1.20)	<0.001	1.52 (1.28, 1.80)	<0.001	−0.09 (−0.18, 0.00)	0.05
Moderate food insecurity (*t*–1)	1.00 (0.99, 1.01)	0.67	1.01 (0.99, 1.03)	0.25	1.12 (1.03, 1.21)	0.006	−0.15 (−0.19, −0.11)	<0.001
Food security (*t*–1)	Referent		Referent		Referent		Referent	
Demographic factors adjusted model^d^								
Severe food insecurity (*t*–1)	1.15 (1.13, 1.17)	<0.001	1.12 (1.08, 1.17)	<0.001	1.41 (1.19, 1.67)	<0.001	−0.05 (−0.14, 0.04)	0.27
Moderate food insecurity (*t*–1)	1.00 (0.99, 1.01)	0.68	1.01 (0.99, 1.03)	0.25	1.06 (0.98, 1.14)	0.16	−0.13 (−0.17, −0.10)	<0.001
Food security (*t*–1)	Referent		Referent		Referent		Referent	
Health status adjusted model^e^								
Severe food insecurity (*t*–1)	1.15 (1.13, 1.17)	<0.001	1.12 (1.08, 1.16)	<0.001	1.30 (1.10, 1.54)	0.002	−0.14 (−0.23, −0.05)	0.003
Moderate food insecurity (*t*–1)	0.99 (0.98, 1.00)	0.02	0.96 (0.94, 0.98)	<0.001	0.97 (0.90, 1.05)	0.50	−0.18 (−0.22, −0.15)	<0.001
Food security (*t*–1)	Referent		Referent		Referent		Referent	
SES adjusted model^f^								
Severe food insecurity (*t*–1)	1.16 (1.14, 1.18)	<0.001	1.24 (1.19, 1.29)	<0.001	1.48 (1.25, 1.75)	<0.001	0.02 (−0.07, 0.11)	0.69
Moderate food insecurity (*t*–1)	1.00 (0.99, 1.01)	0.81	1.04 (1.02, 1.06)	<0.001	1.07 (0.99, 1.16)	0.09	−0.08 (−0.12, −0.05)	<0.001
Food security (*t*–1)	Referent		Referent		Referent		Referent	
Fully adjusted model^g^								
Severe food insecurity (*t*–1)	1.14 (1.12, 1.17)	<0.001	1.18 (1.13, 1.22)	<0.001	1.40 (1.18, 1.65)	<0.001	−0.02 (−0.11, 0.07)	0.67
Moderate food insecurity (*t*–1)	0.99 (0.98, 1.00)	0.02	0.99 (0.98, 1.01)	0.56	1.02 (0.94, 1.11)	0.60	−0.11 (−0.14, −0.07)	<0.001
Food security (*t*–1)	Referent		Referent		Referent		Referent	

Abbreviations: IRR, incidence rate ratio; CI, confidence interval.

^a^ Estimated by random effects Poisson regression.

^b^ Estimated by linear regression.

^c^ Adjusted for year fixed effects.

^d^ Adjusted for age, sex, educational level, marital status, number of household members, region of residence, and year fixed effects.

^e^ Adjusted for chronic conditions (high blood pressure, diabetes, cancer, heart disease, stroke, arthritis), self-rated health, disability, and year fixed effects.

^f^ Adjusted for employment status, household income, and household consumption, and year fixed effects.

^g^ Adjusted for age, sex, educational level, marital status, number of household members, region of residence, chronic conditions (high blood pressure, diabetes, cancer, heart disease, stroke, arthritis), self-rated health, employment status, household income, household consumption, and year fixed effects.

When accounting for all covariates, severe food insecurity was associated with higher incidence rates of outpatient visits (IRR, 1.14; 95% CI, 1.12–1.17), days of hospitalization (IRR, 1.18; 95% CI, 1.13–1.22), and inpatient admissions (IRR, 1.40; 95% CI, 1.18–1.65). Furthermore, moderate food insecurity was associated with a 10.4% (*β* = −0.11; 95% CI, −0.14 to −0.07) or 238,276 KRW reduction in healthcare expenditure. However, moderate food insecurity exhibited no association with outpatient visits (IRR, 0.99; 95% CI, 0.98–1.00), days of hospitalization (IRR, 0.99; 95% CI, 0.98–1.01), or inpatient admissions (IRR, 1.02; 95% CI, 0.94–1.11). Controlling for all covariates shows that moderate food insecurity is not a significant predictor of healthcare utilization at the 5% level. These insignificant results suggest that, in the partially adjusted models moderate food insecurity may capture the effects of food insecurity as well as participants’ sociodemographic characteristics and underlying health status.

## Discussion

In this longitudinal study of representative Korean adults, we found that severe food insecurity was associated with greater subsequent healthcare utilization, including outpatient visits, hospitalizations, and days spent hospitalized. Moderate food insecurity was not associated with healthcare utilization related to hospitalization, indicating a dose-response relationship. The results further showed that moderate food insecurity was associated with 10.4% or 238,276 KRW reductions in personal healthcare expenditures in the subsequent year. The difference between the unadjusted and adjusted models suggests that the unadjusted results were likely confounded by socioeconomic and health variables, and the adjusted results more accurately reflect the true relationship between household food insecurity and healthcare outcomes. Our results support the conceptual frameworks of Andersen and Newman [30] and Becker [29], and replicate previous research showing the complex relationships of household food insecurity with healthcare utilization and expenditure [17, 20, 23, 52, 53].

The findings of this study align with previous research that connects food insecurity to increased healthcare utilization in various contexts. Food insecurity has been associated with greater use of acute healthcare, including emergency department visits, same day surgery, and longer hospital stays in samples of Canadian and American adults [31, 32, 35]. Additionally, food insecure adults seem to use outpatient medical services and pharmaceutical products more frequently than their food secure counterparts [31, 33, 37]. Our analyses contribute to this literature by demonstrating that food insecurity is longitudinally related to outpatient and inpatient medical services, and severe food insecurity leads to greater healthcare utilization in a dose graded manner. Collectively, our results and prior research suggest that food insecure households have a heightened health risk and thus greater needs for medical services [35]. The findings presented here emphasize the importance of considering food insecurity in healthcare reform and interventions.

The negative association between moderate food insecurity and healthcare expenditure could be explained by cost-related underuse of healthcare [27, 28]. Food insecure adults might have switched to more affordable care sources or compromised care quality to reduce the associated financial burden [17]. As a result, they miss opportunities for timely treatment and are more likely to be seen in acute care than their food secure counterparts [54, 55]. The unique healthcare environment in Korea could also contribute to this finding. The NHI program distributes health insurance coverage thinly over a large segment of the population, leading to high out-of-pocket expenses for certain treatments [25]. This co-payment structure can cause vulnerable populations to rely more on primary care and limit the use of specialty medical services for treating severe health conditions. The reform of the national health insurance program to reduce co-payment burdens, currently under consideration by policymakers, may encourage food insecure households to utilize more specialized medical services and thus improve the quality of care received.

It should be noted that the KoWePS targets Korean citizens aged 19 years or older, and therefore food insecurity during childhood and adolescence is not accounted for in this study. Previous research indicates that food insecurity in early life has significant implications for health outcomes and behaviors in adulthood [56–58]. Inadequate nutrition during these critical developmental stages can prompt adaptive bodily responses to food scarcity, which in turn increase the risk of chronic diseases like obesity, diabetes, and heart disease in adulthood [56]. Furthermore, children who grow up in food scarce environments may be more likely to adopt unhealthy eating habits, such as overeating and emotional eating [58]. Those who experienced food insecurity earlier in life are likely to have lower income and unstable job in adulthood, thereby perpetuating the cycle of food insecurity and adverse health outcomes [57]. Collectively, these effects contribute to poor health and an increased demand for healthcare in adulthood. Readers need to be cautious that our results do not fully represent this relationship between food insecurity in adolescence, health outcomes, and health behaviors, and should be interpreted in a restricted context of concurrent health changes that occur when an individual falls into food insecurity during the study period.

The results of this study will contribute to the design of policies and interventions to address food insecurity and healthcare burden among Korean adults. Our findings and prior evidence suggest that the healthcare needs of food-insecure adults may remain unmet [16, 59]. Improving healthcare access requires measures to enhance the income adequacy of vulnerable populations. Policies and programs offering cash assistance have been associated with a lower prevalence of food insecurity [60, 61] and increased use of health services [62]. In Korea, income supplements for working-age adults have been provided by the National Basic Livelihood Security (NBLS), a federal welfare program for citizens below the absolute poverty line. Considering food insecurity in the screening for program qualification may expand the NBLS to food-insecure households and effectively increase their health care-related budgets. Further research is needed to evaluate the effects of income supplementation on healthcare utilization and expenditure among food-insecure Korean adults.

### Strengths and Limitations

Our findings should be interpreted in light of several limitations. The 1-year time gap between surveys may be too short to observe meaningful changes in health status and thus in healthcare utilization and expenditure. If food insecurity alters healthcare use through diet-related diseases or other chronic conditions, it may take longer to observe any relevant changes. Second, sample attrition over time raises the concern that a particular socioeconomic group is over-represented in the study sample. Considering that unhealthy participants are more likely to drop out, it’s plausible to assume that our study sample includes more food secure individuals and those less likely to seek medical services. This non-random attrition could lead to an underestimated coefficient estimate for food insecurity, limiting the representativeness of the regression results in later years. Moreover, a smaller sample size reduces statistical power, thereby increasing the likelihood of Type II errors. Correcting the problem of non-random attrition could result in a larger sample size and stronger associations between food insecurity and healthcare access, which would be identified with lower standard errors. Third, our empirical models could not control for a measure of multimorbidity. The co-existence of multiple chronic conditions can interact with other socioeconomic variables in complex ways, influencing healthcare expenditures non-linearly or modifying the effects of food insecurity. Although our regression models factored in self-rated health, chronic conditions, and disability indicators, the inclusion of multimorbidity could yield a more accurate estimate of the food insecurity effect. Finally, this study lacks data on the quality of care, and thus could not investigate whether and to what extent food insecure households switch to more affordable treatment alternatives. A potential avenue for future research is to explore potential substitution between care options among marginalized populations.

The strength of this study lies in the use of multi-wave, longitudinal, and population-based data. The cohort participating in the baseline survey is sufficiently representative of the working-age population in Korea after the application of sampling weights. The survey design of the KoWePS reduces selection bias and leads to generalizable findings regarding healthcare utilization and expenditure of food-insecure households in Korea. The longitudinal nature of this study is another noteworthy strength as it limits the potential of reverse causality in which excessive healthcare spending leads to food insecurity among low-income households [34]. By lagging the food insecurity indicator, we sought to identify changes in healthcare utilization and expenditure attributable to food insecurity and observe dynamic relationships over 12 years of follow-up. Furthermore, our measure of food insecurity was based on reported experiences of food shortage over the past 12 months, as the USDA guidelines recommend [63]. Most previous studies on healthcare use relied on food insecurity assessments in a 30-day window [20, 21, 23, 32–34, 53, 64], and thus could not disentangle persistent food insecurity from transient disruptions in food supply. This study improves upon the existing literature by linking persistent and chronic experiences of food insecurity to healthcare use in subsequent periods. Lastly, this study was conducted in Korea, where medical services incur large out-of-pocket expenses even for those covered by national health insurance [25]. Our findings might be comparable to studies conducted in the United States and Europe, where food-insecure households with insufficient health insurance coverage are examined.

### Conclusion

This study expands upon prior research by demonstrating the complex relationship between household food insecurity and healthcare utilization and expenditure in working-age adults. Using population-based longitudinal data from Korea and a validated measure of food insecurity, we showed that severe food insecurity was associated with more outpatient visits, hospitalizations, and days spent hospitalized in a dose-response manner. We also found that moderate food insecurity was associated with reductions in personal healthcare expenditure. While the nature of this complex relationship remains unclear, it is evident that food-insecure households constitute a high-risk group. Integrating information on food insecurity status into routine care may help physicians better assess patients’ risk profiles beyond what clinical assessments and diagnoses reveal. Furthermore, policies and programs promoting healthcare access among vulnerable populations need to consider food insecurity when determining priority target groups.
