# Supplementary material for: Food Insecurity, Healthcare Utilization, and Healthcare Expenditures: A Longitudinal Cohort Study
Source: Int J Public Health. 2023 Jul 26;68:1605360. doi: 10.3389/ijph.2023.1605360 (PMC10409992; doi:10.3389/ijph.2023.1605360)
Supplement: Supplementary file 1 [file DataSheet1.pdf]

Supplemental Data

Table S1. Number of observations by survey wave (N=122,241)

Survey wave	Observations (%)
2008	12,166
2009	12,065 (99.2)
2010	11,292 (92.8)
2011	10,475 (86.1)
2012	10,199 (83.8)
2013	9759 (80.2)
2014	9239 (75.9)
2015	8885 (73.0)
2016	8404 (69.1)
2017	8032 (66.0)
2018	7711 (63.4)
2019	7368 (60.6)
2021	6646 (54.6)

Note: Parenthesis presents the retention rate of the follow-up studies relative to the 2009 wave.

Table S2. Association of food insecurity at *t*–1 with health care utilization and expenditures at *t*, demographic factors adjusted model

	Outpatient visits	Inpatient days	Inpatient admissions	Healthcare expenditures
	IRR (95% CI)[a]	IRR (95% CI)[a]	IRR (95% CI)[a]	Adjusted β (95% CI)[b]
Severe food insecurity (*t*–1)	1.15*** (1.13, 1.17)	1.12*** (1.08, 1.17)	1.41*** (1.19, 1.67)	-0.05 (-0.14, 0.04)
Moderate food insecurity (*t*–1)	1.00 (0.99, 1.01)	1.01 (0.99, 1.03)	1.06 (0.98, 1.14)	-0.13*** (-0.17, -0.10)
Age	1.03*** (1.03, 1.03)	1.05*** (1.05, 1.05)	1.02*** (1.02, 1.02)	0.01*** (0.01, 0.01)
Female	1.40*** (1.36, 1.45)	0.63*** (0.58, 0.68)	0.96 (0.91, 1.02)	0.04*** (0.02, 0.07)
High school graduate	0.81*** (0.79, 0.84)	0.81*** (0.75, 0.87)	0.86*** (0.80, 0.92)	0.03 (-0.01, 0.07)
College graduate	0.75*** (0.72, 0.78)	1.03 (0.93, 1.15)	0.82*** (0.75, 0.89)	0.25*** (0.21, 0.29)
Married	1.00 (0.99, 1.00)	0.63*** (0.62, 0.64)	0.95* (0.91, 1.00)	0.31*** (0.28, 0.33)
No. of household members	0.93*** (0.92, 0.93)	1.00 (0.99, 1.00)	0.91*** (0.89, 0.92)	0.16*** (0.16, 0.17)
Urban area	0.94*** (0.93, 0.96)	0.84*** (0.81, 0.87)	1.07** (1.02, 1.14)	-0.05*** (-0.07, -0.02)
Rural area	0.91*** (0.90, 0.93)	0.87*** (0.85, 0.90)	1.12*** (1.06, 1.20)	-0.01 (-0.04, 0.02)

Abbreviations: IRR, incidence rate ratio; CI, confidence interval. Regressions control for year fixed effects. * $p<0.1$, ** $p<0.05$, *** $p<0.01$.

[a] Estimated by random effects Poisson regression.

[b] Estimated by linear regression.

Table S3. Association of food insecurity at t–1 with health care utilization and expenditures at t, health status adjusted model

	Outpatient visits	Inpatient days	Inpatient admissions	Healthcare expenditures
	IRR (95% CI)[a]	IRR (95% CI)[a]	IRR (95% CI)[a]	Adjusted β (95% CI)[b]
Severe food insecurity (t–1)	1.15*** (1.13, 1.17)	1.12*** (1.08, 1.16)	1.30*** (1.10, 1.54)	-0.14*** (-0.23, -0.05)
Moderate food insecurity (t–1)	0.99** (0.98, 1.00)	0.96*** (0.94, 0.98)	0.97 (0.90, 1.05)	-0.18*** (-0.22, -0.15)
High blood pressure	1.31*** (1.31, 1.32)	0.79*** (0.78, 0.80)	0.86*** (0.82, 0.91)	0.05*** (0.03, 0.07)
Diabetes	1.31*** (1.30, 1.32)	0.81*** (0.79, 0.82)	1.00 (0.93, 1.07)	0.08*** (0.05, 0.11)
Cancer	1.20*** (1.18, 1.21)	2.19*** (2.14, 2.23)	3.20*** (2.99, 3.43)	0.68*** (0.63, 0.72)
Heart disease	1.26*** (1.25, 1.27)	0.89*** (0.87, 0.91)	1.33*** (1.23, 1.44)	0.20*** (0.16, 0.24)
Stroke	1.20*** (1.19, 1.22)	1.44*** (1.42, 1.47)	1.26*** (1.16, 1.38)	0.27*** (0.23, 0.32)
Arthritis	1.80*** (1.79, 1.80)	0.99 (0.98, 1.00)	1.05* (1.00, 1.10)	0.15*** (0.12, 0.17)
Good health	1.27*** (1.26, 1.28)	1.29*** (1.25, 1.33)	1.30*** (1.20, 1.41)	0.10*** (0.08, 0.12)
Fair health	1.63*** (1.61, 1.65)	2.28*** (2.21, 2.35)	2.35*** (2.16, 2.56)	0.20*** (0.18, 0.23)
Poor health	1.96*** (1.94, 1.98)	4.61*** (4.47, 4.75)	4.69*** (4.31, 5.11)	0.35*** (0.33, 0.38)
Very poor health	2.14*** (2.12, 2.17)	8.51*** (8.25, 8.78)	8.41*** (7.65, 9.24)	0.58*** (0.53, 0.62)
Disability	1.14*** (1.12, 1.15)	0.82*** (0.80, 0.84)	1.22*** (1.16, 1.30)	-0.06*** (-0.10, -0.03)

Abbreviations: IRR, incidence rate ratio; CI, confidence interval. Regressions control for year fixed effects. * p<0.1, ** p<0.05, *** p<0.01.

[a] Estimated by random effects Poisson regression.

[b] Estimated by linear regression.

Table S4. Association of food insecurity at *t*–1 with health care utilization and expenditures at *t*, socioeconomic status adjusted model

	Outpatient visits	Inpatient days	Inpatient admissions	Healthcare expenditures
	IRR (95% CI)[a]	IRR (95% CI)[a]	IRR (95% CI)[a]	Adjusted β (95% CI)[b]
Severe food insecurity (*t*–1)	1.16*** (1.14, 1.18)	1.24*** (1.19, 1.29)	1.48*** (1.25, 1.75)	0.02 (-0.07, 0.11)
Moderate food insecurity (*t*–1)	1.00 (0.99, 1.01)	1.04*** (1.02, 1.06)	1.07* (0.99, 1.16)	-0.08*** (-0.12, -0.05)
Employed	0.95*** (0.94, 0.95)	0.33*** (0.32, 0.33)	0.38*** (0.36, 0.40)	-0.22*** (-0.24, -0.21)
Self employed	1.03*** (1.02, 1.04)	0.34*** (0.33, 0.34)	0.56*** (0.53, 0.59)	-0.14*** (-0.16, -0.12)
Income Q2	0.96*** (0.95, 0.96)	0.95*** (0.93, 0.96)	0.88*** (0.84, 0.93)	0.04*** (0.01, 0.06)
Income Q3	0.91*** (0.91, 0.92)	0.72*** (0.71, 0.73)	0.64*** (0.59, 0.68)	-0.09*** (-0.12, -0.06)
Income Q4	0.87*** (0.86, 0.88)	0.53*** (0.52, 0.55)	0.48*** (0.45, 0.52)	-0.16*** (-0.19, -0.13)
Income Q5	0.86*** (0.85, 0.87)	0.48*** (0.47, 0.50)	0.41*** (0.38, 0.45)	-0.11*** (-0.15, -0.08)
Consumption Q2	1.06*** (1.05, 1.07)	1.95*** (1.93, 1.98)	1.47*** (1.39, 1.56)	0.52*** (0.49, 0.54)
Consumption Q3	1.13*** (1.12, 1.14)	3.26*** (3.21, 3.32)	1.98*** (1.85, 2.12)	0.81*** (0.78, 0.84)
Consumption Q4	1.16*** (1.15, 1.17)	4.71*** (4.61, 4.80)	2.10*** (1.95, 2.28)	1.06*** (1.03, 1.10)
Consumption Q5	1.24*** (1.22, 1.25)	5.41*** (5.28, 5.54)	2.26*** (2.07, 2.47)	1.35*** (1.32, 1.39)

Abbreviations: IRR, incidence rate ratio; CI, confidence interval. Regressions control for year fixed effects. * $p<0.1$, ** $p<0.05$, *** $p<0.01$.

[a] Estimated by random effects Poisson regression.

[b] Estimated by linear regression.

Table S5. Association of food insecurity at *t*–1 with health care utilization and expenditures at *t*, fully adjusted model

	Outpatient visits	Inpatient days	Inpatient admissions	Healthcare expenditures
	IRR (95% CI)[a]	IRR (95% CI)[a]	IRR (95% CI)[a]	Adjusted β (95% CI)[b]
Severe food insecurity (*t*–1)	1.14*** (1.12, 1.17)	1.18*** (1.13, 1.22)	1.40*** (1.18, 1.65)	-0.02 (-0.11, 0.07)
Moderate food insecurity (*t*–1)	0.99** (0.98, 1.00)	0.99 (0.98, 1.01)	1.02 (0.94, 1.11)	-0.11*** (-0.14, -0.07)
Age	1.02*** (1.02, 1.02)	1.03*** (1.02, 1.03)	1.00 (1.00, 1.00)	0.01*** (0.01, 0.01)
Female	1.35*** (1.31, 1.39)	0.63*** (0.59, 0.68)	0.94** (0.90, 0.99)	0.01 (-0.01, 0.03)
High school graduate	0.85*** (0.83, 0.88)	0.87*** (0.80, 0.94)	0.90*** (0.85, 0.96)	-0.07*** (-0.10, -0.04)
College graduate	0.80*** (0.77, 0.83)	0.94 (0.84, 1.04)	0.89*** (0.82, 0.96)	0.07*** (0.03, 0.11)
Married	1.01** (1.00, 1.02)	0.84*** (0.83, 0.86)	1.03 (0.98, 1.07)	0.24*** (0.22, 0.26)
No. of household members	0.90*** (0.90, 0.91)	0.81*** (0.81, 0.82)	0.81*** (0.80, 0.83)	-0.01 (-0.01, 0.00)
Urban area	0.96*** (0.95, 0.97)	0.94*** (0.91, 0.97)	1.13*** (1.08, 1.19)	-0.03*** (-0.05, -0.01)
Rural area	0.91*** (0.90, 0.93)	1.02 (0.99, 1.06)	1.26*** (1.20, 1.33)	0.06*** (0.03, 0.09)
High blood pressure	1.30*** (1.29, 1.31)	0.80*** (0.79, 0.81)	0.81*** (0.77, 0.86)	0.08*** (0.06, 0.11)
Diabetes	1.30*** (1.29, 1.31)	0.83*** (0.82, 0.85)	0.96 (0.90, 1.03)	0.12*** (0.10, 0.15)
Cancer	1.17*** (1.16, 1.19)	1.86*** (1.82, 1.90)	2.77*** (2.59, 2.97)	0.61*** (0.57, 0.66)
Heart disease	1.25*** (1.24, 1.26)	0.91*** (0.89, 0.93)	1.27*** (1.17, 1.37)	0.23*** (0.20, 0.27)
Stroke	1.19*** (1.17, 1.20)	1.35*** (1.32, 1.37)	1.17*** (1.07, 1.27)	0.28*** (0.24, 0.33)
Arthritis	1.78*** (1.77, 1.79)	0.96*** (0.95, 0.97)	0.99 (0.94, 1.04)	0.17*** (0.15, 0.19)
Good health	1.26*** (1.25, 1.27)	1.26*** (1.23, 1.30)	1.26*** (1.16, 1.36)	0.09*** (0.07, 0.11)
Fair health	1.61*** (1.59, 1.62)	2.19*** (2.13, 2.26)	2.15*** (1.97, 2.35)	0.24*** (0.22, 0.26)
Poor health	1.92*** (1.90, 1.94)	4.10*** (3.97, 4.22)	4.03*** (3.69, 4.41)	0.43*** (0.40, 0.45)
Very poor health	2.10*** (2.07, 2.12)	6.95*** (6.74, 7.18)	6.81*** (6.17, 7.52)	0.64*** (0.60, 0.68)
Disability	1.14*** (1.13, 1.16)	0.87*** (0.85, 0.89)	1.18*** (1.11, 1.25)	0.06*** (0.02, 0.09)
Employed	0.97*** (0.97, 0.98)	0.40*** (0.40, 0.41)	0.58*** (0.55, 0.60)	-0.08*** (-0.10, -0.07)
Self employed	1.04*** (1.03, 1.05)	0.44*** (0.43, 0.44)	0.69*** (0.66, 0.73)	-0.09*** (-0.12, -0.07)
Income Q2	0.99*** (0.98, 1.00)	1.01* (1.00, 1.03)	0.99 (0.93, 1.04)	0.07*** (0.05, 0.10)
Income Q3	0.98*** (0.97, 0.99)	0.86*** (0.85, 0.88)	0.88*** (0.82, 0.95)	0.00 (-0.03, 0.03)
Income Q4	0.97*** (0.96, 0.98)	0.72*** (0.70, 0.74)	0.79*** (0.73, 0.86)	-0.04*** (-0.08, -0.01)
Income Q5	0.99 (0.98, 1.00)	0.75*** (0.72, 0.77)	0.76*** (0.69, 0.84)	0.02 (-0.02, 0.05)

Consumption Q2	1.07***	1.87***	1.55***	0.58***
	(1.07, 1.08)	(1.85, 1.90)	(1.46, 1.64)	(0.55, 0.60)
Consumption Q3	1.17***	3.12***	2.29***	0.92***
	(1.16, 1.18)	(3.06, 3.17)	(2.14, 2.46)	(0.89, 0.95)
Consumption Q4	1.23***	4.43***	2.75***	1.19***
	(1.22, 1.24)	(4.34, 4.53)	(2.54, 2.98)	(1.15, 1.22)
Consumption Q5	1.33***	5.16***	3.10***	1.48***
	(1.31, 1.34)	(5.03, 5.29)	(2.83, 3.40)	(1.45, 1.52)

Abbreviations: IRR, incidence rate ratio; CI, confidence interval. Regressions control for year fixed effects. * $p<0.1$, ** $p<0.05$, *** $p<0.01$.

[a] Estimated by random effects Poisson regression.

[b] Estimated by linear regression.
